# End-of-Induction Response and Tolerability of High-Risk Neuroblastoma Treated with Chemoimmunotherapy—Modified N7 Regimen with Dinutuximab Beta

**DOI:** 10.3390/cancers18061028

**Published:** 2026-03-23

**Authors:** Evelyn R. Lu, Calvin P. L. Hoo, Ho Ming Cheung, I. W. C. Wong, K. F. Kevin Fung, Sylvia L. Y. Chang, Anselm C. W. Lee, Eric C. H. Fu, Dennis T. L. Ku, Jeffrey P. W. Yau, Matthew M. K. Shing, Christy Y. K. Mak, Anthony P. Y. Liu, Godfrey C. F. Chan

**Affiliations:** 1 Department of Paediatrics and Adolescent Medicine, Hong Kong Children’s Hospital, Hong Kong, Chinaanselmcwlee@ha.org.hk (A.C.W.L.); kutl@ha.org.hk (D.T.L.K.); ypwz01@ha.org.hk (J.P.W.Y.); smk423@ha.org.hk (M.M.K.S.); christymyk@hku.hk (C.Y.K.M.);; 2 Department of Paediatrics and Adolescent Medicine, School of Clinical Medicine, LKS Faculty of Medicine, The University of Hong Kong, Hong Kong, China; 3 Nuclear Medicine Unit, Department of Diagnostic and Interventional Radiology, Queen Elizabeth Hospital, Hong Kong, Chinawwc949@ha.org.hk (I.W.C.W.); 4 Department of Radiology, Hong Kong Children’s Hospital, Hong Kong, China; k.fung@ha.org.hk; 5 Neuro-Oncology Section, Hospital for Sick Children, Toronto, ON M5G 1X8, Canada

**Keywords:** high-risk neuroblastoma, anti-GD2, induction chemotherapy, modified N7, immunotherapy, dinutuximab beta

## Abstract

**Background**: The integration of anti-disialoganglioside GD2 (anti-GD2) immunotherapy during induction chemotherapy has emerged as a promising strategy to improve outcomes in high-risk neuroblastoma (HR-NB). This study evaluated the end-of-induction (EOI) response and tolerability of a modified N7 induction regimen combined with dinutuximab beta in a Hong Kong paediatric cohort. **Methods**: A retrospective territory-wide analysis was conducted on nine HR-NB patients treated from 2022 to 2025. They received a modified N7 chemotherapy backbone with dinutuximab beta (17.5 mg/m^2^/day for 4 days per cycle), alongside granulocyte–macrophage colony-stimulating factor (GM-CSF) and low-dose interleukin-2. Response was assessed using the Revised International Neuroblastoma Response Criteria (INRC), and toxicity was graded according to the Common Terminology Criteria for adverse events (CTCAE). **Results**: The EOI objective response rate was 78% (7/9 patients) for the primary tumour site and 100% at metastatic sites. No patient exhibited progressive disease. A modified Curie score of ≤2 on MIBG scan was achieved in 78% of patients. Grade 3 or higher toxicities, including neutropenic fever, enterocolitis, and capillary leak syndrome, were observed in eight patients but were manageable. **Conclusions**: The incorporation of dinutuximab beta into a modified N7 induction regimen demonstrates a satisfactory EOI response rate and a manageable safety profile in children with HR-NB. These preliminary results support the feasibility of this chemoimmunotherapy approach and warrant further investigation in larger cohorts to confirm its efficacy in long-term survival outcomes.

## Simple Summary

High-risk neuroblastoma is an aggressive childhood cancer where intensive induction chemotherapy is a cornerstone of treatment. This study evaluated a novel strategy of integrating a targeted immunotherapy agent, dinutuximab beta, concurrently with a modified induction chemotherapy backbone. The primary aims were to assess the preliminary efficacy of this combination in achieving tumour response and to evaluate its safety and tolerability in a clinical setting. The findings demonstrate a high rate of tumour regression with a manageable adverse event profile, supporting the feasibility of this chemoimmunotherapy approach. This research provides a foundational clinical evidence base that may influence future treatment protocols and justifies larger, confirmatory trials to establish its role in improving survival outcomes for paediatric neuroblastoma patients.

## 1. Introduction

Neuroblastoma, the most common extracranial solid malignancy in children, has an average annual age-standardised incidence of 8 per million in Hong Kong [1]. High-risk disease historically carried a poor prognosis, particularly prior to the advent of anti-disialoganglioside GD2 (anti-GD2) immunotherapy. The introduction of anti-GD2 monoclonal antibodies combined with cytokines such as granulocyte–macrophage colony-stimulating factor (GM-CSF) and interleukin-2 (IL-2) after induction markedly improved outcomes [2,3,4,5,6], elevating 2-year event-free survival (EFS) from 46% to 66% [7,8] and enabling its approval as part of high-risk neuroblastoma (HR-NB) maintenance therapy. Despite this, approximately half of high-risk patients continue to experience significant disease burden due to relapse and refractory disease. Recent efforts have focused on incorporating anti-GD2 antibodies earlier, during induction chemotherapy. Promising phase II data (NB2012 trial) adding hu14.18K322A, a humanised anti-GD2 monoclonal antibody, to the chemotherapy backbone demonstrated improvement in the end-of-induction rate and excellent survival outcomes, with 3-year EFS reaching 73.7% and an overall survival of 86% [9]. Several anti-GD2 monoclonal antibodies, such as dinutuximab, dinutuximab beta [2,10], and naxitamab [11,12], have received regulatory approval and are commercially available for clinical use. The tolerability and preliminary efficacy of incorporating these agents into induction therapy for HR-NB have been documented [3]. A retrospective analysis of 27 patients receiving commercial dinutuximab during induction chemotherapy demonstrated a manageable safety profile and an encouraging end-of-induction response rate [13]. To date, the only available data on upfront chemoimmunotherapy with dinutuximab beta for HR-NB are from a Russian study, which reported that five patients treated with dinutuximab beta combined with a modified GPOH NB2004 protocol experienced acceptable toxicity [14]. Since 2022, commercially available dinutuximab beta has been integrated into the standard induction regimen for HR-NB in Hong Kong, utilising a modified SIOPEN N7 protocol. The existing data from Russia [14], which employed a different chemotherapy backbone, may not be directly applicable to this specific treatment paradigm. Therefore, this study represents the first territory-wide analysis to evaluate the end-of-induction (EOI) response and clinical outcomes in paediatric patients with HR-NB receiving upfront chemoimmunotherapy with dinutuximab beta and modified N7 chemotherapy.

## 2. Materials and Methods

### 2.1. Study Population

This is a pilot study of all paediatric patients with HR-NB treated with chemoimmunotherapy induction, where dinutuximab beta (Qarziba, marketed by Recordati S.p.A., Milan, Italy) was incorporated into the modified N7 backbone, in a territory-wide paediatric oncology centre from 2022 to 2025. All patients were diagnosed with neuroblastoma histologically and categorised as having high-risk, metastatic disease according to the International Neuroblastoma Risk Group Staging System (INRGSS) [15,16]. All patients with incomplete medical records, failure to complete treatment in our centre, or with unclear diagnosis of neuroblastoma were excluded from the review.

### 2.2. Treatment

**Figure 1:**
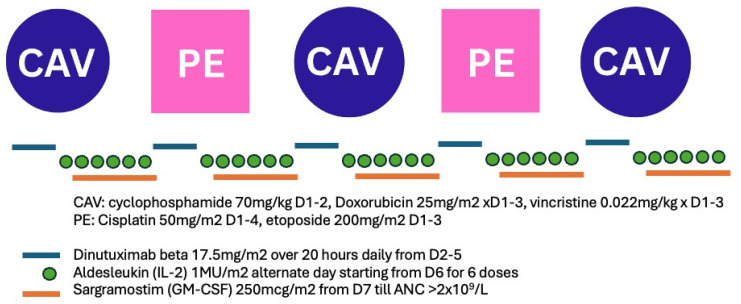
Diagram showing the induction chemotherapy regimen with anti-GD2 antibodies incorporated into the SIOPEN modified N7 regimen.

All high-risk neuroblastoma patients were treated according to the HR-NBL SIOPEN trial protocol, following the modified N7 regimen [2], which consisted of cyclophosphamide, doxorubicin, vincristine, or cisplatin, and etoposide. Dinutuximab beta, a mouse–human chimeric monoclonal IgG1 antibody, produced in a mammalian cell line (CHO) at a dose of 17.5 mg/m^2^/day over 20 h for 4 days with GM-CSF and low-dose IL-2 at 1 MU/m^2^/day, was incorporated into each cycle of chemotherapy (Figure 1). This regimen has been considered “off-label” use and was approved by the hospital’s Drug and Therapeutic Committee. Parents’ consent was sought before commencement of this regimen. Gabapentin premedication was given 3 days before dinutuximab beta and continued until the end of infusion. Intravenous morphine was infused concomitantly with dinutuximab beta to manage neuropathic pain. The modified N7 regimen was chosen for induction chemotherapy based on its more favourable administration schedule. A reduced dinutuximab beta dose of 17.5 mg/m^2^/day was administered, compared to the 20 mg/m^2^/day regimen established in the HR-NBL1 study [2]. This dose modification was implemented due to the postulated increased risk of toxicity when anti-GD2 antibody therapy is combined with chemotherapy. We also implemented it early in the first cycle of induction chemotherapy in the majority of our cases. After induction chemotherapy, patients would undergo definitive surgical resection of the primary tumour followed by single autologous stem cell transplantation using busulfan and melphalan conditioning, then radiotherapy (21 Gy) to the primary tumour. Post-consolidation immunotherapy with five courses of dinutuximab beta at a dose of 20 mg/m^2^/day with GM-CSF and IL-2 was given after irradiation and followed by six cycles of isotretinoin. IL2 was removed from the treatment regimen for the induction and post-consolidation phase for our latest patients (Patients 8, 9) after published results from Ladenstein et al. showing increased toxicity with no efficacy difference [17].

### 2.3. Data Collection

Clinical data, including patient demographics, tumour characteristics, treatment data, outcome data (e.g., end-of-induction response, end-of-treatment response, events and deaths, long-term complications), and toxicity data, were retrieved from electronic patient records. Response assessment was based on the Revised International Neuroblastoma Response Criteria (INRC) [18]. Chemotherapy toxicities were graded according to the National Cancer Institute Common Terminology Criteria for adverse events (CTCAE) [19].

The primary objectives of our study were to evaluate the EOI objective response at the primary tumour site and the metastatic site, the overall response, and the modified Curie score. Secondary outcomes include side effects of the induction regimen, end-of-treatment response, and survival.

### 2.4. Statistical Analysis

Statistical analyses were performed using IBM SPSS Statistics (version 27). Descriptive statistics for patient characteristics and clinical data were presented as medians, ranges, and percentages.

## 3. Results

### 3.1. Patient Characteristics

A cohort of nine patients was identified (Table 1). The median age at diagnosis was 3.5 years (range: 1.5–7.8 years), with a male-to-female ratio of 4:5. Molecular and cytogenetic profiling identified MYCN amplification in two patients and an ALK mutation in one patient; two other patients exhibited segmental chromosomal abnormalities (one with 17q gain, one with partial copy number loss in 1p and 11q). All patients were treated with a modified N7 regimen. Six patients completed five courses of immunotherapy, with the first course initiated concurrently with the first cycle of chemotherapy, and the remaining three patients received four courses starting in the second cycle of chemotherapy.

### 3.2. End-of-Induction (EOI) Response and Toxicity

Disease response was evaluated at the EOI therapy, prior to definitive surgical intervention. Seven patients (78%) exhibited at least a partial response (PR) at the primary site and all patients (100%) achieved a partial response or better at the metastatic sites. Overall, seven patients (78%) achieved a partial response or better in their disease status following induction therapy, with none developing progressive disease (PD). Among the eight patients who underwent surgical resection following induction chemotherapy, pathological evaluation revealed findings of neuroblastoma with treatment response in four patients and the rest (44%) demonstrated complete maturation to ganglioneuroma. Both patients with stable disease at EOI (Patients 4 and 8) exhibited ganglioneuroma on surgical histology. Subsequently, Patient 4 achieved complete treatment remission and remains disease-free for over two years, while Patient 8, who also demonstrated a metastatic complete response at EOI, is currently undergoing post-consolidation immunotherapy.

A modified Curie score of ≤2 on MIBG scans was observed in 78% of patients, which was identified as a prognostic marker in two independent trials [20,21]. Regarding treatment toxicity, eight patients experienced grade 3 or above toxicity, namely neutropenic fever, enterocolitis, mucositis, hypertension and capillary leakage. We did not observe any severe neuropathies in our group. Patient characteristics and their corresponding end-of-induction responses are detailed in Table 1. Five patients in our cohort achieved complete remission. One patient (Patient 3) developed progressive disease following autologous stem cell transplantation. He developed severe veno-occlusive disease at one month post-transplantation with multi-organ involvement. Due to severe pulmonary haemorrhage, the patient was deemed medically unfit for further oncological treatment and ultimately succumbed to respiratory failure. Another patient (Patient 6) developed severe adenoviral pneumonia complicated by acute respiratory distress syndrome at post-transplant, 1.5 months after the second cycle of post-consolidation immunotherapy, necessitating extracorporeal membrane oxygenation (ECMO) support and eventually passed away due to respiratory failure. Patients 3 and 6 died from complications secondary to autologous stem cell transplantation, which were deemed unrelated to the upfront chemoimmunotherapy. Prior to transplantation, both patients had achieved full hematologic recovery and normalised performance status. Patients 8 and 9 had completed induction chemotherapy with recent surgical resection and would undergo autologous stem cell transplantation soon.

## 4. Discussion

Recent evidence supports the efficacy and tolerability of induction chemoimmunotherapy incorporating anti-GD2 antibodies [13,22]. Various combinations of chemotherapy and anti-GD2 antibodies have been extensively investigated [23]. Dinutuximab beta, which is widely adopted outside of North America, is well-established as a maintenance therapy for controlling minimal residual disease [2,24] and is also used in relapsed or refractory neuroblastoma [25,26]. However, limited data exist regarding its use during the induction phase [14]. In this study, we report EOI responses and tolerability in a territory-wide local cohort of nine patients receiving dinutuximab beta in combination with the modified N7 chemotherapy regimen during induction.

Pinto et al. demonstrated that achieving a PR or better at EOI is associated with significantly improved EFS and overall survival, supporting the role of EOI response as a prognostic indicator [27]. In our cohort, the overall response rate of PR or better was 78%, with metastatic sites showing a 100% response rate of PR or above. No patients exhibited progressive disease during induction. Compared to results from the HR-NBL 1.5 trial (*N* = 317) using modified N7 chemotherapy for induction, our cohort demonstrated similar rates of overall response (≥PR: 78% vs. 74%) but higher metastatic site response (≥PR: 100% vs. 86%), alongside a lower incidence of disease progression during induction (0% vs. 1%) [28].

To our knowledge, this study represents the first report on the combination of dinutuximab beta with the modified N7 regimen. Dinutuximab beta was administered at a dose of 17.5 mg/m^2^/day for 4 days, commencing in the first or second cycle of induction chemotherapy, at a lower dose compared to the maintenance dinutuximab beta (20 mg/m^2^/day) in HR-NBL1 [2]. As summarised in Table 2, only a few studies have evaluated anti-GD2 immunotherapy during induction for HR-NB. It is important to note, however, that heterogeneity in response assessment criteria across studies precludes a direct comparative analysis of EOI response rates. The only prior published experience with induction dinutuximab beta came from a Russian study, which used 10 mg/m^2^/day over 5 days, starting in the third cycle, alongside the GPOH NB2004 chemotherapy regimen (containing cisplatin, etoposide, vindesine, vincristine, dacarbazine, ifosfamide, and doxorubicin) for a total of four cycles. The cohort (*N* = 5) reported a 60% PR or above EOI response rate [14]. Despite differences in the chemotherapy backbone, our larger cohort with improved EOI outcomes supports the feasibility and acceptable safety profile of induction chemotherapy with dinutuximab beta. Direct comparisons with other studies are limited by variations in anti-GD2 agents, chemotherapy regimens, and cycle numbers. But importantly, we initiated anti-GD2 therapy earlier (cycle 1 or 2) and administered fewer cycles of chemoimmunotherapy compared with some cohorts that used 5–6 courses or higher anti-GD2 doses (e.g., Furman et al.; Cupit-Link et al.) [9,13]. Even with a lower anti-GD2 dose and fewer cycles, our results did not compromise efficacy or tolerability, underscoring the potential value of early, more concise induction chemoimmunotherapy.

The EOI objective response (≥PR) observed here (78%) was lower than that reported by Furman et al. (93.7%) [9] and Cupit-Link et al. (96%) [13]. This discrepancy is likely attributable to our cohort’s size and a potential underestimation of the primary tumour response by radiological assessment alone, as several cases with residual bulky disease post-induction were found to have mature components upon subsequent surgical resection. In contrast, metastatic site response was excellent (≥PR: 100%), suggesting the overall EOI response may be underestimated. Notable differences in anti-GD2 antibodies (dinutuximab beta vs. hu14.18K322A/dinutuximab) and chemotherapy regimens exist between our study and the two studies mentioned. However, available evidence suggests that dinutuximab beta is non-inferior and may confer superior antibody-dependent cellular cytotoxicity [29,30], making regimen differences an unlikely primary explanation for the lower response rate. Furthermore, the limited global availability of dinutuximab and hu14.18K322A outside North America positions dinutuximab beta as a more accessible alternative.

**Table 2:** Comparison with other studies using anti-GD2 during induction chemotherapy for HR-NB.

Cohort	Patient Number	Tumour Characteristic	Cycles of Anti-GD2	Time of Starting Anti-GD2	Chemotherapy Regime	Name of Anti-GD2 and Dosage	EOI Response
**Induction regimen with induction anti-GD2**							
Hong Kong (current study)	9	MYCN+: 22% ALK+: 11%	4–5	1st or 2nd cycle	Modified N7 (SIOPEN)	Dinutuximab beta 17.5 mg/m^2^/day for 4 days per cycle × 5	≥PR: 78% (Metastatic site: 100%)
Furman et al. (NB2012) [9]	64	MYCN +: 33% ALK+: NA	6	1st cycle	Cyclo+topox2, cisplatin+etoposide alternating with Cyclo+doxo x2	Hu14.18K322A at 40mg/m2/day for 4 days × 6	≥PR: 97%
Cupit-Link et al. [13]	27	MYCN +: 41% ALK+: NA	5–6	1st cycle	Cyclo+topox2, cisplatin+etoposide, cyclo+doxo+VCR, cisplatin+etoposide	Dinutuximab 17.5 mg/m^2^/day for 4 days × 6	≥PR: 96%
Shamanskaya et al. [14]	5	MYCN+: 40% ALK+: 20%	4	3rd cycle	GPOH NB2004	Dinutuximab beta 10 mg/m^2^/day for 5 days × 4	≥PR: 60%
**Other induction regimen without induction anti-GD2**							
Pinto et al. (COG-ANBL-1531) [27]	1242	MYCN+: 44% ALK+: 39%	Nil	Nil	Cyclo+topo x2, cisplatin+etoposide, VCR+doxo+cyclo, cisplatin+etoposide	Nil	≥PR: 79.8%
Garaventa et al. (Rapid COJEC) [28]	313	MYCN+: 39% ALK+: NA	Nil	Nil	Rapid COJEC (cisplatin, carboplatin, etoposide, cyclophosphamide, vincristine)	Nil	≥PR: 70%
Kushner et al. (MSKCC N5) [31]	29	MYCN+: 57% ALK+: NA	Nil	Nil	MSKCC N5 (cyclo, doxo, VCR, etoposide, cisplatin)	Nil	≥PR: 93% (74% in HR-NBL 1.5 study with 317 patients)
Shamanskaya et al. (GPOH NB2004-HR) [32]	151	MYCN+: 50% ALK+: NA	Nil	Nil	N5 (vindesine, cisplatin, etoposide)/N6 (vincristine, dacarbacine, ifosfamide, doxorubicin)	Nil	≥PR: 82.1%
Yoneda et al. (JN-H-07/JN-H-11) [33]	64	MYCN+: 33% ALK+: NA	Nil	Nil	5 cycles of cisplatin, pirarubicin, vincristine, and cyclophosphamide	Nil	≥PR: 67.2%

Abbreviations: ALK+, ALK alteration; cyclo, cyclophosphamide; doxo, doxorubicin; MYCN+, MYCN amplified; topo, topotecan; VCR, vincristine.

This study has several limitations inherent to its design. There is a lack of control group for direct comparison of outcomes with a standard induction regimen without anti-GD2 therapy. Heterogeneity in baseline diagnostic imaging arose due to logistical limitations and variable resource availability, leading a subset of patients undergoing PET-CT rather than diagnostic MIBG scans. All patients demonstrated PET avidity at diagnosis. Although this inconsistency in initial staging modalities posed challenges for standardised disease burden assessment across and within patients, emerging evidence suggests superior sensitivity of PET-CT compared to MIBG scintigraphy in certain clinical contexts [34]. Importantly, this variation did not compromise MIBG EOI response evaluation, which was a proven prognostic indicator alone, as MIBG scans were uniformly performed in all patients at EOI. Minor variations in the number of immunotherapy cycles and the timing of initiation occurred, primarily attributable to logistical and financial considerations. Furthermore, as the integration of anti-GD2 antibodies during induction commenced in Hong Kong in 2022, long-term survival data are not yet mature. Despite the limited cohort size, the observed outcomes are encouraging. Within our cohort, only one patient experienced disease progression, which was attributed to treatment interruption secondary to post-transplant complications. These preliminary results suggest that EOI response may serve as a promising surrogate marker for treatment efficacy in this context and also support the integration of anti-GD2 into induction chemotherapy for the treatment of HR-NB patients.

## 5. Conclusions

In summary, the integration of dinutuximab beta (17.5 mg/m^2^/day for 4 days) with a modified N7 induction regimen demonstrates a satisfactory safety profile and encouraging efficacy, as reflected in the EOI objective response. These preliminary findings support the feasibility of this chemoimmunotherapy approach. However, larger prospective studies with long-term follow-up are required to establish its clinical benefits and survival outcomes robustly.

## Figures and Tables

**Table 1:** Baseline patient characteristics and end-of-induction response.

Patient	Age (Years)	Sex	Primary Site	Metastatic Sites	Curie (Dx)	MYCN	ALK	SCA	No. of Anti-GD2 Courses	Primary Tumour Response	Metastatic Site Response	Curie (EOI)	Overall Response	Toxicity	Outcome	FU Period (Months)
1	2.7	M	Left adrenal	Bone, BM, LN	NA	Neg	Pos	Neg	5	CR	CR	0	CR	Gr 2 neutropenic colitis, Gr 3 neutropenic fever	CR1	31
2	6.6	M	Retroperitoneum	Bone, BM, LN	17	Neg	NA	17q gain	4	PR	PR	7	PR	Nil	CR1	35
3	2.3	M	Right adrenal	Bone, LN	4	Pos	NA	Neg	5	PR	CR	1	PR	Gr 3 hypertension, Gr 3 capillary leak	PD, Death	10
4	5.2	F	Posterior mediastinum	Bone, BM, LN	4	Neg	Neg	Neg	5	SD	PR	2	MR	Gr 3 neutropenic fever	CR1	27
5	4.5	F	Left adrenal	Bone, BM, LN	20	Neg	NA	Partial loss of 1p and 11q	5	PR	CR	2	PR	Gr 3 neutropenic fever	CR1	24
6	3.5	M	Retroperitoneum	Bone, LN	3	Neg	Neg	Neg	5	PR	CR	1	PR	Gr 3 neutropenic colitis	Death	11
7	7.8	F	Left adrenal	Bone, BM, LN, Liver, spine	NA	Neg	NA	Neg	5	PR	PR	10	PR	Gr 3 mucositis, Gr 3 neutropenic fever	CR1	12
8	2.6	F	Posterior mediastinal	Bone, LN, spine	NA	Neg	NA	Neg	4	SD	CR	1	MR	Gr 3 neutropenic fever	NA	6
9	1.6	F	Left adrenal	Bone, BM, LN	NA	Pos	NA	Neg	4	PR	PR	1	PR	Gr 3 neutropenic fever	NA	5

Abbreviations: BM, bone marrow; CR, complete response; CR1, complete remission; Curie, modified Curie score; Dx, at diagnosis; EOI, at end-of-induction; F, female; FU, follow-up; Gr, grade; LN, lymph node; M, male; MR, minor response; NA, not available; Neg, negative; PD, progressive disease; Pos, positive; PR, partial response; SCA, segmental chromosomal aberrations; SD, stable disease.

## Data Availability

The raw data supporting the conclusions of this article will be made available by the authors on request.

## Abbreviations

The following abbreviations are used in this manuscript:

ALK	Anaplastic lymphoma kinase
BM	Bone marrow
COJEC	Rapid COJEC chemotherapy
CR	Complete response
CR1	Complete remission
Curie	Modified Curie score
Cyclo	Cyclophosphamide
Doxo	Doxorubicin
Dx	At diagnosis
EFS	Event-free survival
EOI	At end-of-induction
F	Female
FU	Follow-up
LN	Lymph node
M	Male
MIBG	Metaiodobenzylguanidine
MR	Minor response
MYCN	MYCN proto-oncogene
N7	Modified N7 chemotherapy
NA	Not available
Neg	Negative
N	Number of patients
OS	Overall survival
PD	Progressive disease
Pos	Positive
PR	Partial response
SD	Stable disease
Topo	Topotecan
